# Supplementary material for: Time-series transcriptome analysis identified differentially expressed genes in broiler chicken infected with mixed Eimeria species
Source: Front Genet. 2022 Aug 8;13:886781. doi: 10.3389/fgene.2022.886781 (PMC9393255; doi:10.3389/fgene.2022.886781)
Supplement: Supplementary file 2 [file DataSheet1.ZIP › 7dpi_KEGG.Gsea.1625115961950/KEGG_CYTOKINE_CYTOKINE_RECEPTOR_INTERACTION.html]

Details for gene set KEGG\_CYTOKINE\_CYTOKINE\_RECEPTOR\_INTERACTION[GSEA]

|  || Dataset | TMM\_7dpi\_gct\_format\_7dpi\_gct\_format.Class\_4dpi.cls #PC\_versus\_NC.Class\_4dpi.cls #PC\_versus\_NC\_repos |
| Phenotype | Class\_4dpi.cls#PC\_versus\_NC\_repos |
| Upregulated in class | 0 |
| GeneSet | KEGG\_CYTOKINE\_CYTOKINE\_RECEPTOR\_INTERACTION |
| Enrichment Score (ES) | -0.43124723 |
| Normalized Enrichment Score (NES) | -1.590275 |
| Nominal p-value | 4.1076198E-4 |
| FDR q-value | 0.07914807 |
| FWER p-Value | 0.7268 |
Table: GSEA Results Summary

  

Fig 1: Enrichment plot: KEGG\_CYTOKINE\_CYTOKINE\_RECEPTOR\_INTERACTION      
 Profile of the Running ES Score & Positions of GeneSet Members on the Rank Ordered List

  

| SYMBOL | TITLE | RANK IN GENE LIST | RANK METRIC SCORE | RUNNING ES | CORE ENRICHMENT || 1 | CXCL13 | na | 126 | 1.176 | 0.0053 | No |
| 2 | BMP2 | na | 134 | 1.164 | 0.0205 | No |
| 3 | CXCR5 | na | 365 | 0.867 | 0.0128 | No |
| 4 | TNFRSF8 | na | 628 | 0.673 | -0.0001 | No |
| 5 | TNFRSF21 | na | 675 | 0.648 | 0.0048 | No |
| 6 | CXCR1 | na | 701 | 0.635 | 0.0113 | No |
| 7 | CCL26 | na | 704 | 0.634 | 0.0197 | No |
| 8 | TNFRSF11B | na | 717 | 0.626 | 0.0272 | No |
| 9 | IL11RA | na | 1136 | 0.473 | -0.0016 | No |
| 10 | NGFR | na | 1188 | 0.459 | 0.0003 | No |
| 11 | CCL4 | na | 1209 | 0.453 | 0.0047 | No |
| 12 | CXCL12 | na | 1235 | 0.446 | 0.0086 | No |
| 13 | IL1B | na | 1237 | 0.445 | 0.0146 | No |
| 14 | INHBB | na | 1569 | 0.362 | -0.0084 | No |
| 15 | CD40LG | na | 1613 | 0.353 | -0.0072 | No |
| 16 | TNFRSF6B | na | 1943 | 0.280 | -0.0311 | No |
| 17 | TGFB2 | na | 1954 | 0.278 | -0.0282 | No |
| 18 | PDGFC | na | 2266 | 0.227 | -0.0513 | No |
| 19 | TNFSF13B | na | 2307 | 0.222 | -0.0517 | No |
| 20 | TNFRSF1B | na | 2342 | 0.215 | -0.0517 | No |
| 21 | CCL28 | na | 2640 | 0.171 | -0.0743 | No |
| 22 | IL9R | na | 2743 | 0.158 | -0.0808 | No |
| 23 | ACVR1 | na | 3013 | 0.125 | -0.1017 | No |
| 24 | TNFRSF10B | na | 3036 | 0.122 | -0.1019 | No |
| 25 | MET | na | 3037 | 0.122 | -0.1003 | No |
| 26 | AMHR2 | na | 3241 | 0.094 | -0.1161 | No |
| 27 | INHBA | na | 3410 | 0.073 | -0.1293 | No |
| 28 | CCL19 | na | 3544 | 0.059 | -0.1397 | No |
| 29 | IL6R | na | 3564 | 0.056 | -0.1405 | No |
| 30 | IL22RA2 | na | 3786 | 0.030 | -0.1587 | No |
| 31 | CCR7 | na | 4067 | -0.003 | -0.1822 | No |
| 32 | IL18R1 | na | 4237 | -0.023 | -0.1962 | No |
| 33 | TNFRSF1A | na | 4239 | -0.023 | -0.1959 | No |
| 34 | PDGFB | na | 4266 | -0.026 | -0.1978 | No |
| 35 | PDGFA | na | 4308 | -0.031 | -0.2008 | No |
| 36 | CRLF2 | na | 4344 | -0.035 | -0.2033 | No |
| 37 | OSMR | na | 4598 | -0.060 | -0.2238 | No |
| 38 | TNFRSF13B | na | 4829 | -0.084 | -0.2420 | No |
| 39 | CNTF | na | 4893 | -0.092 | -0.2461 | No |
| 40 | PRLR | na | 4924 | -0.095 | -0.2473 | No |
| 41 | EDA | na | 4999 | -0.103 | -0.2521 | No |
| 42 | VEGFC | na | 5000 | -0.103 | -0.2507 | No |
| 43 | IL20RA | na | 5127 | -0.115 | -0.2598 | No |
| 44 | IL15 | na | 5256 | -0.127 | -0.2688 | No |
| 45 | CX3CL1 | na | 5266 | -0.127 | -0.2679 | No |
| 46 | TGFB1 | na | 5297 | -0.130 | -0.2686 | No |
| 47 | CSF1 | na | 5321 | -0.133 | -0.2688 | No |
| 48 | CD40 | na | 5477 | -0.147 | -0.2798 | No |
| 49 | RELT | na | 5480 | -0.147 | -0.2780 | No |
| 50 | ACVRL1 | na | 5963 | -0.197 | -0.3159 | No |
| 51 | TGFBR2 | na | 6133 | -0.218 | -0.3272 | No |
| 52 | LEPR | na | 6235 | -0.228 | -0.3326 | No |
| 53 | IL12RB1 | na | 6273 | -0.232 | -0.3326 | No |
| 54 | BMP7 | na | 6319 | -0.236 | -0.3332 | No |
| 55 | IFNAR2 | na | 6336 | -0.238 | -0.3313 | No |
| 56 | IL4R | na | 6643 | -0.269 | -0.3534 | No |
| 57 | FLT4 | na | 6800 | -0.286 | -0.3627 | No |
| 58 | PDGFRA | na | 6935 | -0.300 | -0.3699 | No |
| 59 | TNFRSF25 | na | 6939 | -0.301 | -0.3661 | No |
| 60 | IL1RAP | na | 6944 | -0.302 | -0.3624 | No |
| 61 | FASLG | na | 6950 | -0.302 | -0.3587 | No |
| 62 | GHR | na | 7374 | -0.349 | -0.3896 | No |
| 63 | HGF | na | 7414 | -0.353 | -0.3881 | No |
| 64 | TGFBR1 | na | 7506 | -0.362 | -0.3909 | No |
| 65 | IFNGR2 | na | 7611 | -0.373 | -0.3946 | No |
| 66 | TNFRSF4 | na | 7712 | -0.386 | -0.3978 | No |
| 67 | IL6ST | na | 7807 | -0.397 | -0.4003 | No |
| 68 | CCR5 | na | 7839 | -0.400 | -0.3975 | No |
| 69 | TNFSF8 | na | 7845 | -0.400 | -0.3925 | No |
| 70 | TNFRSF18 | na | 7874 | -0.405 | -0.3894 | No |
| 71 | CXCR4 | na | 7900 | -0.408 | -0.3860 | No |
| 72 | CNTFR | na | 8279 | -0.455 | -0.4116 | No |
| 73 | ACVR1B | na | 8355 | -0.465 | -0.4117 | No |
| 74 | IL1R1 | na | 8525 | -0.487 | -0.4193 | No |
| 75 | TNFRSF14 | na | 8668 | -0.509 | -0.4244 | Yes |
| 76 | IL7 | na | 8686 | -0.511 | -0.4189 | Yes |
| 77 | PDGFRB | na | 8726 | -0.517 | -0.4152 | Yes |
| 78 | IL18RAP | na | 8727 | -0.517 | -0.4082 | Yes |
| 79 | EDA2R | na | 8734 | -0.517 | -0.4017 | Yes |
| 80 | VEGFA | na | 8816 | -0.526 | -0.4014 | Yes |
| 81 | IL2RG | na | 8882 | -0.534 | -0.3996 | Yes |
| 82 | CSF1R | na | 8919 | -0.538 | -0.3954 | Yes |
| 83 | CSF2RA | na | 8987 | -0.547 | -0.3936 | Yes |
| 84 | CSF3R | na | 9044 | -0.555 | -0.3908 | Yes |
| 85 | BMPR1A | na | 9126 | -0.569 | -0.3899 | Yes |
| 86 | CX3CR1 | na | 9141 | -0.571 | -0.3834 | Yes |
| 87 | KDR | na | 9221 | -0.586 | -0.3821 | Yes |
| 88 | TNFSF11 | na | 9243 | -0.589 | -0.3759 | Yes |
| 89 | IL15RA | na | 9275 | -0.595 | -0.3705 | Yes |
| 90 | VEGFD | na | 9472 | -0.627 | -0.3785 | Yes |
| 91 | IFNAR1 | na | 9530 | -0.636 | -0.3747 | Yes |
| 92 | TGFB3 | na | 9555 | -0.639 | -0.3680 | Yes |
| 93 | CSF2RB | na | 9787 | -0.672 | -0.3784 | Yes |
| 94 | ACVR2A | na | 9906 | -0.693 | -0.3790 | Yes |
| 95 | CCR4 | na | 10029 | -0.717 | -0.3795 | Yes |
| 96 | TNFSF10 | na | 10126 | -0.736 | -0.3776 | Yes |
| 97 | CCL20 | na | 10167 | -0.744 | -0.3709 | Yes |
| 98 | EDAR | na | 10276 | -0.765 | -0.3697 | Yes |
| 99 | CCL17 | na | 10285 | -0.768 | -0.3600 | Yes |
| 100 | CCR6 | na | 10297 | -0.770 | -0.3505 | Yes |
| 101 | IL21R | na | 10343 | -0.779 | -0.3437 | Yes |
| 102 | TNFRSF19 | na | 10354 | -0.781 | -0.3340 | Yes |
| 103 | CCR8 | na | 10388 | -0.788 | -0.3261 | Yes |
| 104 | CCR2 | na | 10404 | -0.791 | -0.3167 | Yes |
| 105 | IL18 | na | 10446 | -0.798 | -0.3093 | Yes |
| 106 | LIFR | na | 10540 | -0.818 | -0.3061 | Yes |
| 107 | TNFRSF11A | na | 10604 | -0.834 | -0.3001 | Yes |
| 108 | IL10RB | na | 10676 | -0.848 | -0.2946 | Yes |
| 109 | IL2RA | na | 10774 | -0.872 | -0.2910 | Yes |
| 110 | PLEKHO2 | na | 10843 | -0.891 | -0.2846 | Yes |
| 111 | IL1R2 | na | 10937 | -0.917 | -0.2800 | Yes |
| 112 | CCL5 | na | 10974 | -0.929 | -0.2705 | Yes |
| 113 | KITLG | na | 10999 | -0.936 | -0.2599 | Yes |
| 114 | TNFSF15 | na | 11007 | -0.938 | -0.2478 | Yes |
| 115 | IL10RA | na | 11062 | -0.955 | -0.2394 | Yes |
| 116 | CXCL14 | na | 11098 | -0.963 | -0.2293 | Yes |
| 117 | TNFRSF9 | na | 11139 | -0.977 | -0.2194 | Yes |
| 118 | FLT1 | na | 11183 | -0.995 | -0.2096 | Yes |
| 119 | IL17RA | na | 11188 | -0.996 | -0.1965 | Yes |
| 120 | MPL | na | 11201 | -1.000 | -0.1839 | Yes |
| 121 | EGFR | na | 11415 | -1.085 | -0.1872 | Yes |
| 122 | IL7R | na | 11582 | -1.161 | -0.1855 | Yes |
| 123 | XCL1 | na | 11583 | -1.161 | -0.1697 | Yes |
| 124 | BMPR2 | na | 11698 | -1.238 | -0.1626 | Yes |
| 125 | IL13RA1 | na | 11716 | -1.255 | -0.1470 | Yes |
| 126 | CCL1 | na | 11719 | -1.260 | -0.1302 | Yes |
| 127 | IL12RB2 | na | 11728 | -1.267 | -0.1137 | Yes |
| 128 | IL22 | na | 11774 | -1.308 | -0.0998 | Yes |
| 129 | KIT | na | 11813 | -1.348 | -0.0847 | Yes |
| 130 | XCR1 | na | 11841 | -1.394 | -0.0681 | Yes |
| 131 | FAS | na | 11850 | -1.412 | -0.0497 | Yes |
| 132 | IL2RB | na | 11878 | -1.457 | -0.0323 | Yes |
| 133 | IFNGR1 | na | 11917 | -1.547 | -0.0145 | Yes |
| 134 | FLT3 | na | 11939 | -1.644 | 0.0060 | Yes |
Table: GSEA details [plain text format]

  

Fig 2: KEGG\_CYTOKINE\_CYTOKINE\_RECEPTOR\_INTERACTION      
 Blue-Pink O' Gram in the Space of the Analyzed GeneSet

  

Fig 3: KEGG\_CYTOKINE\_CYTOKINE\_RECEPTOR\_INTERACTION: Random ES distribution      
 Gene set null distribution of ES for **KEGG\_CYTOKINE\_CYTOKINE\_RECEPTOR\_INTERACTION**
